# Severe Rectal Syphilis in the Setting of Profound HIV Immunosuppression: A Case Report Highlighting ERG/CD38 Immunophenotyping and a Review of the Literature

**DOI:** 10.3390/idr17040085

**Published:** 2025-07-16

**Authors:** Diana Marcela Carmona Valencia, Juan Diego López, Shirley Vanessa Correa Forero, Diana Marcela Bonilla Bonilla, Jorge Karim Assis, Yamil Liscano

**Affiliations:** 1 Specialization in Internal Medicine, Department of Health, Universidad Santiago de Cali, Cali 760035, Colombia; diana.carmona00@usc.edu.co (D.M.C.V.); juan.lopez39@usc.edu.co (J.D.L.); svcorreaf@gmail.com (S.V.C.F.); diana.bonilla01@usc.edu.co (D.M.B.B.); 2 Department of Research and Education, Clínica de Occidente S.A., Santiago de Cali 760046, Colombia; investigacion@clinicadeoccidente.com; 3 Grupo de Investigación en Salud Integral (GISI), Departamento Facultad de Salud, Universidad Santiago de Cali, Cali 760035, Colombia

**Keywords:** rectal syphilis, proctitis, HIV infection, *Treponema pallidum*, ERG immunohistochemistry, CD38 immunostaining

## Abstract

**Background and Aim:** Syphilis, caused by *Treponema pallidum*, classically presents with genital or anal chancres; rectal involvement is rare and frequently misdiagnosed as inflammatory bowel disease or malignancy. We describe an unusually severe case of syphilitic proctitis in the setting of advanced HIV-related immunosuppression (CD4 39 cells/µL), in which targeted immunophenotyping (ERG and CD38) was a valuable adjunctive tool in the differential diagnosis. **Case Presentation:** A 46-year-old man with a recent history of erosive gastritis and esophageal candidiasis presented after six months of unintentional 20 kg weight loss, profound fatigue, intermittent fevers, profuse diarrhea, and two episodes of hematemesis. Workup revealed a new diagnosis of HIV infection (CD4: 39 cells/µL; viral load: 87,837 copies/mL). Contrast-enhanced CT demonstrated uniform, concentric rectal wall thickening (“target sign”). Colonoscopic biopsy showed exuberant granulation tissue and dense plasma cell infiltrates. Immunohistochemistry revealed a dense infiltrate of CD38-positive plasma cells and ERG-positive endothelial proliferation. These findings, in the context of positive serology, were highly supportive of a spirochetal etiology and helped differentiate it from potential mimics. Serology was positive for latent late syphilis (VDRL 1:64). The patient received three weekly doses of intramuscular benzathine penicillin; lumbar puncture excluded neurosyphilis. **Discussion:** This is among the first reported cases of syphilitic proctitis in a patient with CD4 < 50 cells/µL, where advanced immunophenotyping differentiated syphilitic inflammation from neoplastic or inflammatory mimics. Profound immunosuppression accelerates disease progression and yields atypical clinical features. **Conclusion:** In HIV-infected patients with chronic rectal symptoms, especially those with CD4 < 50 cells/µL, syphilitic proctitis must be considered. Integration of radiologic assessment, histopathology with ERG/CD38 staining, and serologic testing permits prompt diagnosis. Early benzathine penicillin therapy and rigorous clinical and serologic follow-up are essential to prevent complications, including neurosyphilis.

## 1. Introduction

Syphilitic proctitis is a rare form of secondary infection by *Treponema pallidum (T. pallidum)*, presenting most often in men who have sex with men (MSM) and individuals coinfected with HIV [1,2]. Clinically, it mimics other causes of proctitis, such as inflammatory bowel disease, lymphogranuloma venereum, and even rectal neoplasms, leading to frequent misdiagnoses and delays in appropriate therapy [3,4,5]. The pathophysiology involves spirochetal invasion of the rectal mucosa, resulting in concentric wall thickening, mucosal ulceration, and a dense plasmacytic infiltrate on histology [4,6].

From an epidemiological standpoint, syphilis incidence has risen sharply worldwide over the past decade. In Colombia, approximately 20,000 new HIV cases and 300 syphilis cases per 100,000 population are reported annually, though data on rectal involvement remain scarce. A cross-sectional study in Medellín found *T. pallidum* prevalence of 16.7 percent among MSM, but notably 2.2 percent in the general population and 1.4 percent in women, underscoring that syphilitic proctitis can occur outside classic risk groups. This broader epidemiology mandates vigilance even in patients without overt risk factors [3,7,8].

Diagnosing syphilitic proctitis requires a multimodal approach: demonstration of *T. pallidum*—specific serology (e.g., VDRL, TPPA), imaging studies revealing the characteristic “target sign” of rectal wall enhancement on contrast-enhanced CT or MRI, endoscopic evaluation showing erythema, exudates, and ulcerations—and supportive histopathology, where immunohistochemical stains such as ERG and CD38 can help characterize the inflammatory infiltrate and aid in the differential diagnosis. In immunocompromised hosts, particularly those with CD4 counts <50 cells/µL, disease progression is accelerated, and presentations may include systemic features like significant weight loss, fever, and gastrointestinal bleeding, further complicating the clinical picture [2,4,9].

To our knowledge, this is the first reported case of syphilitic proctitis in Colombia in a patient with advanced HIV (CD4 = 39 cells/µL), in which detailed immunophenotyping (ERG/CD38) and high-resolution imaging were pivotal for diagnosis. The use of targeted immunohistochemistry to distinguish spirochetal inflammation from other etiologies is especially novel in our setting, where such advanced diagnostics remain limited. This case underscores the imperative for increased access to multidisciplinary expertise, combining infectious diseases, gastroenterology, radiology, and pathology, and to promote advanced imaging and histopathologic techniques for timely identification and treatment of functional rectal infections.

## 2. Case Presentation

### 2.1. History and Presentation

A 46-year-old MSM, with a past medical history of erosive antral gastritis and esophageal candidiasis (diagnosed by upper endoscopy in July 2024 and treated with fluconazole), presented with a six-month history of progressive symptoms. He reported unintentional weight loss of approximately 20 kg, profound fatigue and weakness, two episodes of hematemesis, reduced appetite, intermittent fevers up to 38.5 °C, more than ten diarrheal stools per day, diffuse abdominal pain, and frequent lightheadedness.

### 2.2. Key Timeline of Events

**Figure 1:**
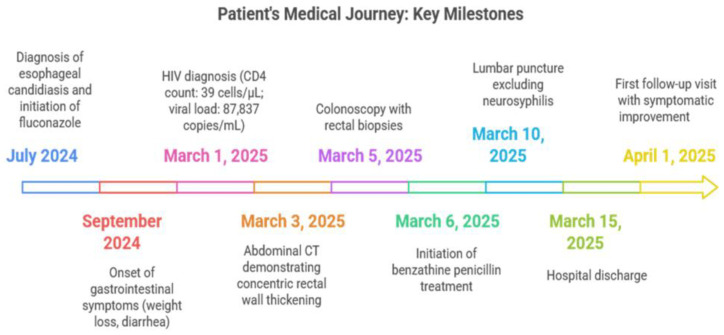
Patient’s medical journey: key milestones.

Figure 1 summarizes the patient’s course from esophageal candidiasis treated in July 2024 to the onset of weight loss and diarrhea in September 2024, followed by an HIV diagnosis on 1 March 2025. Imaging on March 3 revealed rectal wall thickening, leading to colonoscopy and biopsy on March 5, initiation of benzathine penicillin on March 6, and lumbar puncture excluding neurosyphilis on March 10. The patient was discharged on March 15 and showed clinical and serological improvement at follow-up on 1 April 2025.

### 2.3. Initial Evaluation

On admission, upper gastrointestinal bleeding was initially suspected. However, the history of esophageal candidiasis, rare in otherwise healthy adults, prompted evaluation for immunosuppression. HIV testing returned positive, staging him as CDC stage 3 with a CD4 count of 39 cells/µL and a viral load of 87,837 copies/mL. The initial laboratory evaluation was critical, revealing a moderate normocytic anemia (Hgb: 9.2 g/dL), severe lymphopenia (Absolute Lymphocyte Count: 0.50 × 10^3^/µL), and a significant inflammatory state (CRP: 145 mg/L). Notably, renal function, liver enzymes, and coagulation studies were within normal limits.

### 2.4. Imaging and Endoscopic Findings

**Figure 2:**
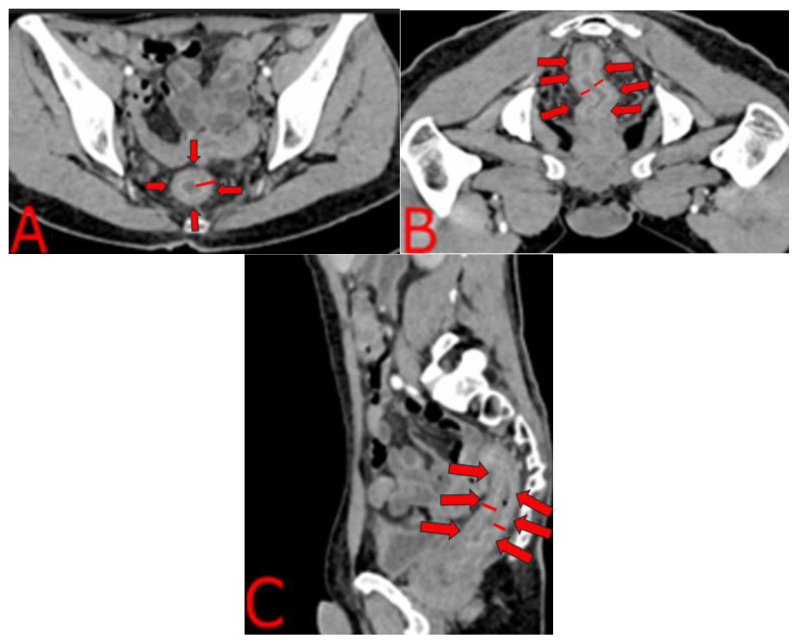
Concentric rectal wall thickening on contrast-enhanced CT. (**A**) Axial image at the mid-pelvic level demonstrates uniform, concentric thickening of the rectal wall, with preservation of the fat plane and no discrete mass. The wall measures up to 8 mm in thickness and shows homogeneous enhancement. (**B**) A higher axial slice just above the elevator ani reveals continuous involvement of the distal rectum, without perirectal fat stranding or lymphadenopathy. (**C**) Sagittal reformatted image illustrates the full cranio-caudal extent of rectal involvement, from the rectosigmoid junction down to the anal canal, maintaining a smooth, stratified appearance.

The contrast-enhanced CT findings are highly characteristic of an inflammatory proctitis rather than a neoplastic or ischemic process. Figure 2A shows uniform, circumferential thickening of the rectal wall, measuring up to 8 mm, with smooth margins and preserved perirectal fat, while Figure 2B demonstrates that this concentric involvement extends continuously just above the elevator ani without accompanying lymphadenopathy or fat stranding. The sagittal reconstruction in Figure 2C confirms the full cranio-caudal reach of the abnormality, from the rectosigmoid junction to the anal canal, exhibiting the classic “target sign” of mucosal hyperenhancement and submucosal hypodensity. This layered enhancement pattern, reflecting mucosal hyperemia and submucosal edema, strongly suggests an infectious or inflammatory etiology. In our immunocompromised patient with confirmed *T. pallidum* infection, these CT features were highly suggestive of a severe infectious or inflammatory etiology rather than a discrete neoplastic mass. Recognizing this radiologic signature prompted urgent serologic testing and a directed endoscopic biopsy. The positive serology results allowed for the immediate initiation of antibiotic therapy, while the biopsy was crucial for excluding malignancy and later confirming the diagnosis.

**Figure 3:**
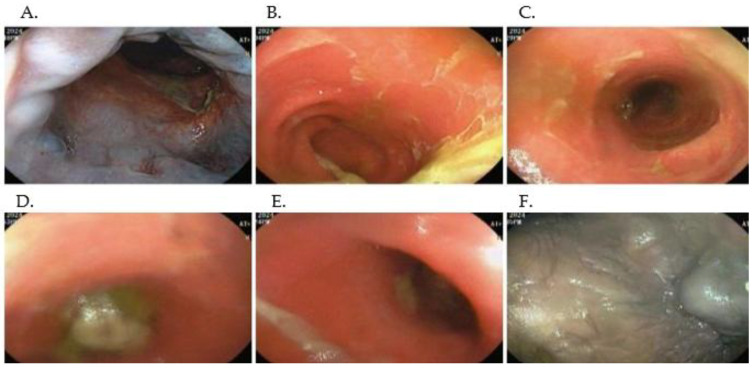
Representative colonoscopic findings. (**A**) Anal canal. Congested, erythematous mucosa with superficial erosions and overlying yellow-white exudate. (**B**) Distal transverse colon. Diffuse mucosal hyperemia with adherent fecaloid debris and mild edema. (**C**). Descending colon. Patchy pseudomembranous plaques on an erythematous, friable background. (**D**). Mid-transverse colon. Scattered superficial ulcerations with granulation tissue and thick adherent exudate. (**E**) Sigmoid colon. Circumferential erythema, loss of normal vascular pattern, and mucosal friability. (**F**) Perianal skin (anoderm). Bluish-black hematoma with adjacent mucosal edema and granularity.

Guided by the CT findings of diffuse, concentric rectal wall thickening (Figure 2), a full colonoscopic evaluation was performed. This revealed multifocal fecal impaction extending to the mid-transverse colon, which limited complete mucosal assessment (Figure 3B–D). The rectal and sigmoid mucosa appeared diffusely erythematous, with adherent yellow-white exudative membranes and pseudomembranous plaques (Figure 3A–C). Prominent edema and friability were noted throughout, punctuated by scattered superficial ulcerations and granulation tissue in the mid-transverse and sigmoid segments (Figure 3D–E). Examination of the anoderm identified an external hematoma surrounded by edematous, granular mucosa (Figure 3F). Targeted biopsies from these areas demonstrated granulation tissue with abundant capillaries, a dense plasma cell infiltrate, and occasional eosinophils, with complete loss of normal colonic architecture, findings that, in conjunction with the imaging pattern, confirmed an inflammatory proctitis of infectious etiology.

### 2.5. Microbiological and Histopathological Workup

**Figure 4:**
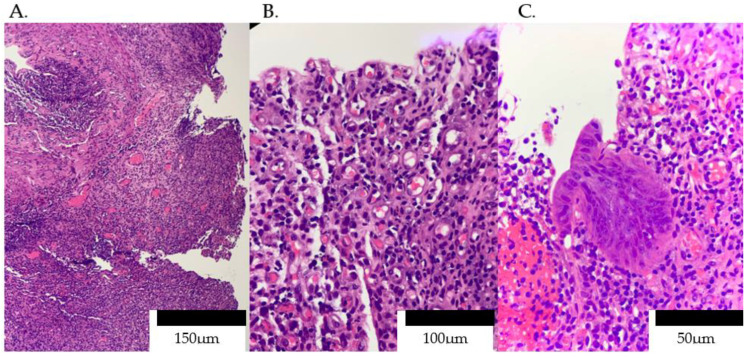
Granulation tissue with numerous capillaries permeated by dense plasma cells and occasional eosinophils. (**A**) Low-power (×40) view showing exuberant granulation tissue replacing normal mucosa, with numerous newly formed capillaries and a dense mononuclear infiltrate. (**B**) Intermediate magnification (×200) highlights small, thin-walled capillaries lined by plump endothelial cells, surrounded by a heavy infiltrate of plasma cells. (**C**) High-power (×400) detail of sheets of mature plasma cells admixed with occasional eosinophils, and focal epithelial regeneration without granuloma formation.

In light of the CT and endoscopic findings suggestive of diffuse proctitis (Figure 2 and Figure 3), we performed an extensive opportunistic infection panel, which returned negative results for hepatotropic viruses, serum and CSF cryptococcal antigen, tuberculin skin testing, urinary lipoarabinomannan, and Toxoplasma serology. In contrast, *T. pallidum* serology was strongly positive, with a VDRL titer of 1:64, and the absence of cutaneous or genital lesions supported a diagnosis of secondary syphilis. Targeted immunohistochemical studies further refined the diagnosis: CMV and HSV-2 stains were negative, whereas endothelial cells demonstrated diffuse ERG positivity, and the dense inflammatory infiltrate stained uniformly for CD38 (Figure 4). While not specific, these histopathologic findings were crucial. The dense, CD38-positive plasma cell infiltrate is a classic hallmark of the host response to syphilis. The ERG-positive capillary proliferation, seen in this context, helped differentiate the process from other conditions like Kaposi’s sarcoma. Therefore, when combined with the positive serology and clinical presentation, this histopathologic pattern provided strong supportive evidence for a diagnosis of syphilitic proctitis and helped to effectively exclude other opportunistic or neoplastic etiologies.

### 2.6. Treatment and Prophylaxis

The patient received three weekly intramuscular injections of benzathine penicillin (2.4 million IU each). A lumbar puncture ruled out neurosyphilis. Prophylaxis against opportunistic pathogens was initiated with trimethoprim/sulfamethoxazole (160/800 mg on Monday, Wednesday, and Friday) and weekly azithromycin (3 × 500 mg). In accordance with standard syndromic management for infectious proctitis and while awaiting further microbiological results, empiric doxycycline was administered to cover common co-pathogens, such as *Chlamydia trachomatis* [10].

### 2.7. Additional Investigations and Management

New respiratory symptoms prompted chest CT, which showed centrilobular micronodules with a tree-in-bud pattern and peripheral ground-glass opacities in upper and lower lobes bilaterally. Bronchoscopy with bronchoalveolar lavage excluded opportunistic pulmonary infections. Ophthalmologic evaluation revealed inferior retinal edema and posterior vitreous detachment in the right eye, suggestive of ocular toxoplasmosis; brain MRI excluded CNS involvement. Intravenous trimethoprim/sulfamethoxazole (15 mg/kg/day divided every eight hours) and oral prednisolone (25 mg daily for two weeks), along with topical prednisolone drops, were administered.

### 2.8. Antiretroviral Therapy and Outcome

Combination antiretroviral therapy with tenofovir, emtricitabine, and dolutegravir was initiated. Over the subsequent days, the patient’s gastrointestinal symptoms markedly improved, and he was discharged in stable condition.

## 3. Discussion

### 3.1. Epidemiology and Clinical Significance

Syphilitic proctitis is an uncommon manifestation of secondary T. pallidum infection, most frequently reported in MSM and individuals with HIV coinfection [11,12]. However, its occurrence in other populations underscores the need for heightened clinical suspicion even when classic risk factors are absent. Moreover, national surveillance data from Colombia report approximately 20,000 new HIV diagnoses each year (incidence 39.2 per 100,000) and an overall syphilis incidence of ~300 per 100,000, figures driven largely by antenatal screening programs, with scant data on the broader population [1,3,7,8,13,14,15].

### 3.2. Clinical Presentation and Differential Diagnosis

In immunocompromised patients, particularly those with advanced HIV, syphilitic infection can progress rapidly and present with nonspecific anorectal symptoms: pain, tenesmus, purulent discharge, or rectal bleeding. Moreover, nearly 30% of cases may be asymptomatic, further complicating timely identification [16]. In our patient, the six-month history of weight loss, low-grade fever, and severe diarrhea, coupled with the CT and colonoscopy findings (Figure 2 and Figure 3), highlighted the complexity of distinguishing syphilitic proctitis from other etiologies. Notably, the concentric rectal wall thickening on CT (Figure 2) and the diffuse erythema with pseudomembranous exudates on endoscopy (Figure 3) might initially have suggested an IBD flare or malignancy. This case reinforces the importance of including syphilis in the differential diagnosis of chronic proctitis, especially in immunosuppressed individuals [16,17].

### 3.3. Comparative Case Analysis

In this comprehensive comparison (Table 1 and Table 2), our case (“This work, 2025”) stands out in several respects. First, our patient presented with advanced immunosuppression (CD4 39 cells/µL) and a six-month symptom duration, which contrasts with the shorter courses reported by Díaz-Jaime et al. (2017) [18], who described relatively acute presentations (1–2 weeks) in patients on suppressive ART. Similarly, Pisani et al. (2015) [19] and Chen (2021) [13] noted symptom durations of only a few weeks to two months. The protracted six-month evolution in “This work” likely contributed to more extensive mucosal involvement and systemic complications (hematemesis, profound weight loss), underscoring the importance of early recognition, especially in severely immunocompromised hosts.

Clinical manifestations in our patient were more multisystemic, including fatigue, hematemesis, high-volume diarrhea, and intermittent fevers, whereas most previously reported rectal syphilis cases in MSM (e.g., Kobayashi et al., 2012 [20]; Afzal et al., 2024 [1]) focused on localized anorectal symptoms (bleeding, tenesmus, pain). The patient’s hematemesis, while a confounding factor in the initial workup, is not a typical feature of syphilitic proctitis. A more plausible etiology is the patient’s documented history of erosive gastritis and esophageal candidiasis, rather than a direct manifestation of syphilis. This broader symptom complex may reflect synergy between HIV-related immunodeficiency and disseminated *T. pallidum* infection, a feature less commonly documented in earlier series.

Imaging and endoscopic features in our case were remarkably distinctive. We observed a classic “target sign” on contrast-enhanced CT of diffuse, concentric rectal wall thickening with layered enhancement and preserved fat planes, which proved pathognomonic for inflammatory rather than neoplastic etiologies. In contrast, prior reports (e.g., Pisani et al., 2015 [19]) described mass-like lesions with lymphadenopathy mimicking T3N+ rectal cancer on MRI/PET–CT, and Afzal et al. (2024) [1] noted only mild rectal wall thickening on CT without the characteristic target appearance. Our detailed radiologic description thus adds a valuable imaging correlate to the literature that can guide clinicians toward correct diagnosis.

Histopathologically, all cases share a plasma-cell-rich infiltrate, but our work further demonstrates ERG positivity in proliferating capillaries and uniform CD38 expression in plasma cells (Figure 4), refining previous Warthin–Starry or Steiner staining techniques. These immunohistochemical hallmarks provide a more specific tissue signature for syphilitic proctitis, beyond the nonspecific chronic inflammation and spirochete visualization in earlier biopsies (Díaz-Jaime et al., 2017 [18]; Hunter et al., 2025 [14]).

Regarding treatment and outcomes, our patient received the standard benzathine penicillin G regimen and adjunctive prophylaxis for opportunistic infections. Unlike some earlier cases that employed alternative antibiotics (Hunter et al., 2025 [14] used doxycycline) or single-dose penicillin (Díaz-Jaime et al., 2017 [18]; Figueroa et al., 2018 [21]), our multimodal approach, including ART initiation, led to rapid symptomatic and endoscopic resolution by one month and robust serologic improvement by follow-up. This underscores the necessity of integrating comprehensive antimicrobial therapy, HIV management, and supportive prophylaxis in advanced HIV-associated syphilitic proctitis.

Compared with previously published rectal syphilis cases, typically characterized by shorter symptom duration, well-controlled HIV, and localized anorectal findings, our work highlights a more severe, multisystem presentation in a profoundly immunosuppressed patient, delineates pathognomonic imaging and immunohistochemical features, and demonstrates the effectiveness of an integrated treatment strategy. These distinctions reinforce the importance of considering syphilitic proctitis in the differential diagnosis of chronic proctitis, particularly among patients with advanced HIV, and of utilizing targeted radiologic, histopathologic, and therapeutic approaches to optimize outcomes.

### 3.4. Diagnostic Workup and Histopathology

A comprehensive evaluation for opportunistic infections was negative for hepatotropic viruses, cryptococcal antigen, mycobacterial antigens, and Toxoplasma (Section 2.5). Serologic testing revealed a VDRL titer of 1:64, confirming syphilis despite the absence of cutaneous lesions. Histopathology was critical: biopsies demonstrated exuberant granulation tissue with ERG-positive capillaries and a dense CD38-positive plasma cell infiltrate without granulomas (Figure 4). These immunohistochemical findings are highly specific for syphilitic inflammation and effectively exclude mycobacterial or fungal etiologies. To further enhance diagnostic sensitivity, particularly in cases where spirochetes are sparse or atypical, molecular testing via PCR for *T. pallidum* on tissue specimens can be invaluable. By directly detecting treponemal DNA, PCR assays offer rapid, objective confirmation of infection and can guide appropriate therapy even when histologic or serologic results are equivocal.

### 3.5. Management and Follow-Up

Treatment of syphilitic proctitis follows standard guidelines for late latent syphilis, with intramuscular benzathine penicillin (3 doses of 2.4 million IU weekly) remaining the regimen of choice, even in HIV-coinfected patients [2,9,11] Our patient tolerated therapy without adverse reactions and underwent lumbar puncture to exclude neurosyphilis, in accordance with recommendations for CD4 < 50 cells/µL [2,10]. Close serological monitoring is essential to document four-fold decreases in titers and to detect relapse, which is more common in the immunosuppressed [5,10]. To complement laboratory follow-up, integrating patient-reported outcome measures (PROMs) into clinical visits could provide valuable insights into quality of life and symptom resolution over time, fostering a more patient-centered approach and potentially identifying subtle relapses or treatment-related complications earlier.

### 3.6. Lessons Learned and Future Directions

This case illustrates that:

A high index of suspicion is required for rectal syphilis across diverse patient populations.

Multimodal diagnosis, combining imaging (Figure 2 and Figure 3), histopathology (Figure 3), and serology, is vital to avoid misdiagnosis.

Standard penicillin therapy remains effective, but rigorous follow-up is paramount.

Perhaps the most crucial lesson from this case is the impact of the diagnostic delay. The patient first presented with esophageal candidiasis in July 2024, a sentinel opportunistic infection that should have prompted immediate investigation for underlying immunodeficiency. The six-month gap between this initial presentation and the eventual diagnosis allowed for the relentless progression of both HIV and syphilis, culminating in the severe, multisystemic illness observed upon admission. This underscores a critical public health message: the presence of an opportunistic infection like esophageal candidiasis in an adult without other known risk factors (e.g., inhaled steroid use) must be considered an indication for immediate HIV screening. Instituting such a practice could prevent life-threatening complications and significantly alter the disease course for many patients.

Future research should prioritize the validation of advanced molecular diagnostics, such as tissue-based *T. pallidum* PCR assays, to enhance sensitivity and specificity in challenging biopsy specimens, particularly when histopathology or serology yields equivocal results. Additionally, the implementation of telemedicine platforms for remote serologic monitoring could improve long-term follow-up in resource-limited settings, facilitating timely detection of treatment failure or relapse. Moreover, establishing a prospective, multicenter registry of syphilitic proctitis cases would enable systematic collection of clinical, radiologic, histopathologic, treatment, and outcome data, thereby allowing researchers to identify predictors of prognosis, optimize management protocols, and standardize care pathways across diverse healthcare environments [22,23,24,25].

## 4. Conclusions

Syphilitic proctitis should be considered in any immunocompromised patient, particularly those with advanced HIV, presenting with chronic or multisystem anorectal symptoms. Recognition of the characteristic “target sign” on contrast-enhanced CT (diffuse, concentric wall thickening with layered enhancement) can distinguish inflammatory proctitis from malignancy and prompt directed biopsy. Histopathology, supported by immunohistochemical characterization of the infiltrate (e.g., with CD38 and ERG to aid in the differential diagnosis), provides crucial supportive data. However, the definitive diagnosis rests on the combination of clinical findings, positive serology, and response to therapy. Treatment with standard intramuscular benzathine penicillin, alongside opportunistic infection prophylaxis and initiation of antiretroviral therapy, leads to rapid clinical and serologic resolution; incorporating patient-reported outcome measures can further guide follow-up.

Looking forward, establishing a prospective registry of syphilitic proctitis cases across multiple centers will enable systematic evaluation of presentation patterns, imaging and histologic signatures, treatment regimens, and outcomes. Parallel efforts to validate tissue-based *T. pallidum* PCR assays and deploy telemedicine platforms for remote serologic monitoring will enhance diagnostic sensitivity and improve long-term care, particularly in resource-limited settings.

## Figures and Tables

**Table 1:** Comparative overview of rectal syphilis case reports.

Author (Year)	Location	Age/Sex	Clinical Background	HIV Status	Symptom Duration
This work (2025)	Universidad Santiago de Cali & Clínica de Occidente, CALI, CO	46 M (MSM)	Erosive antral gastritis; esophageal candidiasis (2024)	New HIV (CD4 39 cells/µL; VL 87,837 copies/mL)	6 months
Díaz-Jaime et al. (2017) [18]	La Fe Hospital, Valencia, Spain	35 M (MSM)	Known HIV; no other comorbidities	Not specified	2 weeks
Pisani et al. (2015) [19]	Univ. of Milan, Milan, Italy	48 M (MSM)	No prior history; monogamous MSM relationship	Not specified	Few weeks
Kobayashi et al. (2012) [20]	Tokyo Univ. Hosp., Tokyo, Japan	26 M (MSM)	Newly diagnosed HIV at presentation	CD4 227 cells/µL; not on ART	Several weeks
Figueroa et al. (2018) [21]	Specialized Ctr., Lima, Peru	53 M (MSM)	No known HIV until workup; prior painless chancre	New HIV CD4 275 cells/µL	~3 months
Chen and Wang (2021) [13]	Municipal Hosp., Hangzhou, China	31 M (MSM)	Newly diagnosed HIV; multiple anonymous partners	HIV−1 Ab +; CD4% 15.9%	~2 months
Hunter I et al. (2025) [14]	Vancouver, Canada	60 s M	Previously healthy; condomless receptive anal sex with casual partner	HIV−; negative serology	>3 months
Afzal Z et al. (2024) [1]	Cambridge, UK	64 M	Diverticular disease; multiple MSM partners (elicited later)	HIV−; negative serology	Several weeks

MSM: Men who have sex with men; M: Male; HIV: Human immunodeficiency virus; Ab: Antibody.

**Table 2:** Comparative overview of reported rectal syphilis case reports.

Author (Year)	Key Manifestations	Biopsy Highlight	Imaging Brief	Serology	Treatment
This work (2025)	Wt loss, ↑diarrhea, hematemesis, fever, pain	ERG^+^ capillaries; CD38^+^ plasma cells	CT “target sign”; colonoscopy: exudates, ulcers	Trep-EIA^+^; VDRL 1:64	PCN G IM × 3 wk + TMP/SMX and AZM + Dox + ART
Díaz-Jaime et al. (2017) [18]	Hematochezia, rectal mass	Plasma-cell-rich; spirochetes (WS stain)	Endoscopic ulcer only	TPHA^+^; RPR^+^	PCN G IM (single dose)
Pisani et al. (2015) [19]	Rectal bleeding; palpable 3 cm mass	WS stain + spirochetes	Colon ulcer; MRI T3N+; PET FDG^+^	Trep^+^; HSV/CMV/HHV-8-	PCN G 2.4 M IU IM × 1
Kobayashi et al. (2012) [20]	Anal pain, bleeding, palm/sole rash	Deep ulcer; no organisms on H&E	Endoscopic deep ulcer	FTA-ABS^+^; RPR NS	IV PCN G (dose unspecified)
Figueroa et al. (2018) [21]	Tenesmus, bleeding, pain, lymphadenopathy	Granulomatous infiltrate; spirochetes	Deep ulcer + multiple erosions	VDRL–; FTA-ABS^+^	PCN G IM × 1 + ART
Chen and Wang (2021) [13]	Tenesmus, mucous discharge, rash	Plasma cell infiltrate; *T. pallidum* IHC^+^	Sigmoidoscopy: superficial ulcers	TPPA 1:1280; RPR 1:256	PCN G IM × 3 wk + ART
Hunter I et al. (2025) [14]	Verrucous lesions, pain, bleeding	Psoriasiform hyperplasia; ERG^+^, CD38^+^	HRA: friable, hyperaemic mass	EIA^+^; TPPA^+^; RPR 1:4096	Doxy 100 mg BID × 14 d
Afzal Z et al. (2024) [1]	Bowel habit change; mass; cutaneous lesions	Chronic proctitis; spirochetes (Steiner)	CT: mild thickening + nodes; MRI NL	EIA^+^; TPPA^+^; RPR 1:128	Oral PCN G course (14 d)

Wt loss: weight loss; ↑ diarrhea: increased diarrhea; Hematemesis: vomiting of blood; PCN G: penicillin G; IM: intramuscular; wk: week; TMP/SMX: trimethoprim/sulfamethoxazole; AZM: azithromycin; Dox: doxycycline; ART: antiretroviral therapy; WS stain: Warthin–Starry stain; CT: computed tomography; MRI: magnetic resonance imaging; PET FDG: positron emission tomography with fluorodeoxyglucose; FTA-ABS: fluorescent treponemal antibody absorption test; RPR: rapid plasma reagin; TPHA: *T. pallidum* hemagglutination assay; Trep EIA: treponemal enzyme immunoassay; TPPA: *T. pallidum* particle agglutination assay; H&E: hematoxylin and eosin stain; IHC: immunohistochemistry; HRA: high-resolution anoscopy; NS: not stated; NL: normal; BID: twice daily; d: days.

## Data Availability

Data are contained within the article.

## Abbreviations

The following abbreviations are used in this manuscript:

Ab	antibody
ART	antiretroviral therapy
AZM	azithromycin
BID	twice daily
CD4	cluster of differentiation 4
CSF	cerebrospinal fluid
CT	computed tomography
Dox	doxycycline
d	days
EIA	enzyme immunoassay
ERG	ETS-related gene
FTA ABS	fluorescent treponemal antibody absorption test
H&E	hematoxylin and eosin stain
HRA	high-resolution anoscopy
HIV	human immunodeficiency virus
IBD	inflammatory bowel disease
IHC	immunohistochemistry
IM	intramuscular
M	male
MRI	magnetic resonance imaging
MSM	men who have sex with men
NL	normal
NS	not stated
PCN G	penicillin G
PET FDG	positron emission tomography with fluorodeoxyglucose
RPR	rapid plasma reagin
TMP/SMX	trimethoprim/sulfamethoxazole
TPHA	*Treponema pallidum* hemagglutination assay
Trep EIA	treponemal enzyme immunoassay
TPPA	*Treponema pallidum* particle agglutination assay
VDRL	Venereal Disease Research Laboratory
WS stain	Warthin–Starry stain
Wt loss	weight loss
↑ diarrhea	increased diarrhea
hematemesis	vomiting of blood
wk	week
